# **Anthrax** Lethal Toxin-Mediated Disruption of Endothelial VE-Cadherin Is Attenuated by Inhibition of the Rho-Associated Kinase Pathway

**DOI:** 10.3390/toxins3101278

**Published:** 2011-10-20

**Authors:** Jason M. Warfel, Felice D’Agnillo

**Affiliations:** 1 Laboratory of Biochemistry and Vascular Biology, Division of Hematology, Center for Biologics Evaluation and Research, Food and Drug Administration, Bethesda, Maryland, MD 20892, USA; Email: jason.warfel@fda.hhs.gov; 2 Department of Microbiology and Immunology, Georgetown University Medical Center, Washington, DC 20057, USA

**Keywords:** anthrax lethal toxin, vascular endothelium, adherens junction, barrier function, cadherin, actin stress fibers

## Abstract

Systemic anthrax disease is characterized by vascular leakage pathologies. We previously reported that anthrax lethal toxin (LT) induces human endothelial barrier dysfunction in a cell death-independent manner with actin stress fiber formation and disruption of adherens junctions (AJs). In the present study, we further characterize the molecular changes in the AJ complex and investigate whether AJ structure and barrier function can be preserved by modulating key cytoskeletal signaling pathways. Here, we show that LT reduces total VE-cadherin protein and gene expression but the expression of the key linker protein beta-catenin remained unchanged. The changes in VE-cadherin expression correlated temporally with the appearance of actin stress fibers and a two-fold increase in phosphorylation of the stress fiber-associated protein myosin light chain (p-MLC) and cleavage of Rho-associated kinase-1 (ROCK-1). Co-treatment with ROCK inhibitors (H-1152 and Y27632), but not an inhibitor of MLC kinase (ML-7), blocked LT-induced p-MLC enhancement and stress fiber formation. This was accompanied by the restoration of VE-cadherin expression and membrane localization, and attenuation of the LT-induced increase in monolayer permeability to albumin. Together, these findings suggest the ROCK pathway may be a relevant target for countering LT-mediated endothelial barrier dysfunction.

## 1. Introduction

Inhalational anthrax is a disease caused by inhaling spores of the gram-positive bacterium *Bacillus anthracis*. Many of the symptoms of systemic inhalational anthrax can be attributed to the action of anthrax toxin, which is made up of three secreted proteins: protective antigen (PA) and lethal factor (LF), which combine to form lethal toxin (LT), and edema factor (EF) which combines with PA to form edema toxin [1,2]. PA binds to the cell surface receptors ANTXR1 and ANTXR2, leading to endocytosis of the enzymatic moieties EF and LF [3]. Once in the cytosol, EF is a calcium/calmodulin-dependent adenylate cyclase, causing accumulation of the secondary signaling molecule cAMP [4]. LF is a zinc metalloprotease that cleaves proteins of the MEK family, disrupting mitogen-activated protein kinase (MAPK) signaling [5,6]. While both receptors are ubiquitously expressed, ANTXR2 is considered the dominant receptor *in vivo* by virtue of its 11-fold higher affinity for PA [7].

Systemic anthrax infection is often accompanied by profound vascular pathologies including edema, hemorrhage, pleural effusion, and vasculitis in animals and humans [8,9,10,11]. Importantly, pleural effusions and endothelial pathologies are also observed in animals treated with purified LT [12,13,14]. Toxin receptor expression also appears to be enriched on the endothelium [15]. These findings have supported the idea that LT may directly target the endothelium during systemic anthrax infection, when serum levels of LF and PA can exceed 200 and 1000 ng/mL respectively [16,17,18,19]. In addition, LT was shown to increase vascular permeability in a zebrafish model in the absence of endothelial cell death, suggesting that LT may dysregulate endothelial junctions *in vivo* [20]. This finding is consistent with recent studies in LT-treated mice showing increased leakage of intravenous fluorescent dyes in the lung [21]. 

Consistent with the vascular pathologies of anthrax, we previously reported that LT induces cell death-independent barrier dysfunction in primary human lung microvascular endothelial cell culture characterized by actin stress fiber formation and altered adherens junction (AJ) structure [22]. VE-cadherin, the major component of AJs, is a single-span transmembrane protein that is unique to endothelial cells and promotes homophilic interaction between neighboring cells [23]. Importantly, the cytoplasmic tail of VE-cadherin is linked to the actin cytoskeleton via scaffolding catenin proteins. In quiescent endothelium, the actin cytoskeleton plays a critical role in regulating the endothelial barrier by providing stability for AJs. Here, we further characterize the effects of LT on AJ integrity and investigate whether AJ structure and barrier function can be preserved by modulating key cytoskeletal regulating pathways. The present findings suggest that LT-induced barrier dysfunction and AJ structure may be rescued in part by inhibiting the Rho-associated kinase (ROCK) pathway.

## 2. Materials and Methods

### 2.1. Reagents

Phosphate-buffered saline (PBS) and Hank’s balanced salt solution with calcium and magnesium (HBSS) were obtained from Invitrogen (Carlsbad, CA, USA). The MLC kinase (MLCK) inhibitor ML-7, the ROCK inhibitors H-1152 (Rki) and Y-27632 (Y27) were purchased from EMD Chemicals (Gibbstown, NJ, USA). LF, PA, and inactive mutant LF_E687C_ were kindly provided by Dr. Stephen H. Leppla (National Institutes of Health, Bethesda, MD) [24,25]. Toxin proteins were diluted in sterile PBS before cell treatment. All other reagents were purchased from Sigma Chemical Co. (St. Louis, MO, USA).

### 2.2. Antibodies

Goat polyclonal antibodies to VE-cadherin (catalog #sc-6458) and ROCK-1 (C-19) (catalog #sc-6055), and rabbit polyclonal antibodies to ROCK-1 (H-85) (catalog #sc-5560), ROCK-2 (catalog #sc-5561), beta-catenin (catalog #sc-7199) and tubulin (catalog #sc-9104) were purchased from Santa Cruz Biotechnology (Santa Cruz, CA, USA). Rabbit polyclonal antibody to p-MLC (Thr18/Ser19) (catalog #3674) was purchased from Cell Signaling Technology (Danvers, MA, USA). Rabbit polyclonal antibody to MEK1 (catalog #07-641) was obtained from Millipore (Billerica, MA, USA). 

### 2.3. Endothelial Cell Culture and Treatment

Primary human lung microvascular endothelial cells were obtained from Lonza (Walkersville, MD, USA) and cultured as described previously [22]. For inhibitor experiments, confluent monolayers were pretreated with the specified inhibitor for 30 min unless otherwise indicated. Without washing out inhibitor, cells were treated with LT (100 ng/mL LF and 500 ng/mL PA) or inactive mutant LT (100 ng/mL LF_E687C_ and 500 ng/mL PA). Individual toxin components LF or PA did not alter endothelial morphology or barrier function [22].

### 2.4. Albumin Permeability Assay

Cells grown to confluence on porous membrane inserts (12 mm diameter, 0.4 µm pore size) were treated as described above. After 72 h, 50 µL of culture medium from the upper chamber was replaced with an equal amount of medium containing 5 mg/mL FITC-HSA (final concentration 500 µg/mL). After 2 h, 20 µL samples were drawn from the lower chamber and diluted 10-fold. Data were collected from duplicate inserts per treatment in each experiment. Fluorescence measurements were obtained using a microplate reader (Genios™, Tecan, Research Triangle Park, NC, USA) with excitation and emission filters of 485 and 535 nm, respectively. FITC-HSA concentrations were calculated using a FITC-HSA standard curve. To quantify the trans-membrane flux (μg/h/cm^2^), the FITC-HSA concentration was multiplied by the volume of the lower chamber and divided by the membrane area and the FITC-HSA incubation time.

### 2.5. Immunocytochemistry

Cells were grown to confluence in 24-well dishes, and treated with LT in the presence or absence of inhibitors as indicated. Monolayers were fixed with 3.7% paraformaldehyde, washed, and blocked in HBSS containing 10% goat serum. Monolayers were incubated with anti-phospho-MLC (1:100) and anti-VE-cadherin (1:200) for 1 h at room temperature. Immunofluorescence analysis using Alexa Fluor 555-labeled secondary antibodies (1:800 dilution) and Alexa Fluor 488-labeled phalloidin (1:100 dilution) to stain filamentous actin (Invitrogen) was performed as described previously [22]. Nuclei were stained with Hoechst 33342 (5 µg/mL). Phosphorylated MLC and VE-cadherin were quantified as described previously [26]. Briefly, images of 4-6 independent fields were taken from duplicate treatment wells stained with either secondary antibody alone or primary and secondary antibodies. The mean gray value was calculated from each field using ImageJ software (NIH, Bethesda, MD, USA) and averaged for each group. The average mean gray value for the secondary antibody alone group was subtracted from the average mean gray value from the primary and secondary antibodies-stained group to obtain the fluorescence intensity (adjusted mean gray value).

### 2.6. Real-Time PCR

RNA extraction and gene expression procedures were described previously [27]. Gene expression was analyzed using TaqMan gene expression assays for *CDH5* (VE-cadherin), *GAPDH*, and *CTNNB1* (beta-catenin). Reactions were performed in triplicate and run on the Applied Biosystems 7900HT real-time PCR system. Fold gene expression was calculated using the 2^-∆∆C^T method using *GAPDH* as the reference gene [27].

### 2.7. Preparation of Whole Cell Extracts

Whole cell extracts were prepared as described previously using ice cold RIPA lysis buffer (50 mM Tris, 150 mM NaCl, 1% IgePal-630, 1 mM EDTA, 0.25% sodium deoxycholate) containing protease inhibitors (Cocktail Set III, Calbiochem, CA) and the phosphatase inhibitors NaF (1 mM), sodium orthovanadate (1 mM), sodium pyrophosphate (2.5 mM), and beta glycerophosphate (1 mM) [28]. 

### 2.8. Western Blotting

Reduced samples were run on NuPAGE 4-12% gradient Bis-Tris gels in MOPS SDS running buffer. Proteins were transferred to PVDF membranes and detected as described previously [28]. Densitometry analysis was performed using Image J software.

### 2.9. Statistical Analysis

Data are represented as means ± SE for replicate experiments. Statistical analysis was performed by ANOVA with *post-hoc* Student’s *t*-test using the JMP (version 7) software (SAS Institute Inc, Cary, NC, USA). *p* < 0.05 was considered statistically significant.

## 3. Results

### 3.1. LT Reduces VE-Cadherin Protein and mRNA Expression

**Figure 1:**
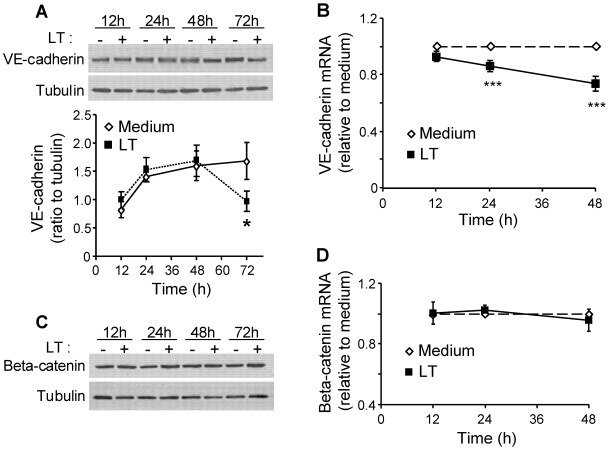
Lethal toxin (LT) reduces expression of VE-cadherin but not beta-catenin. Cells were treated with medium (-) or LT for indicated time points. Whole cell lysates were analyzed by Western blot for VE-cadherin (**A**) or beta-catenin (**C**). Blots were reprobed for tubulin as a loading control. The graph in A represents Western blot densitometry for VE-cadherin normalized to tubulin (*n* = 4). RNA was collected and analyzed for (**B**) VE-cadherin and (**D**) beta-catenin transcript relative to GAPDH by real-time PCR. Data presented as means ± SE (*n* = 3). * *p* < 0.05 *versus* medium; *** *p* < 0.001 *versus* medium.

We previously reported that LT disrupts AJ integrity in endothelial cells [22]. To further characterize this AJ dysfunction, we analyzed the protein and mRNA expression of VE-cadherin and beta-catenin. LT reduced total VE-cadherin protein expression by 40% compared to non-treated control cells at 72 h (Figure 1A). These data were consistent with a reduction in VE-cadherin mRNA at 24 and 48 h (Figure 1B). Treatment with LF, PA, or the combination of an inactive mutant LF and PA had no effect on VE-cadherin expression (data not shown). Meanwhile, LT had no effect on protein or mRNA expression of beta-catenin (Figure 1C,D). These findings suggest that LT-induced disruption of AJs primarily involves alterations in VE-cadherin expression.

### 3.2. LT Induces MLC Phosphorylation that Is Attenuated by ROCK Inhibitors

**Figure 2:**
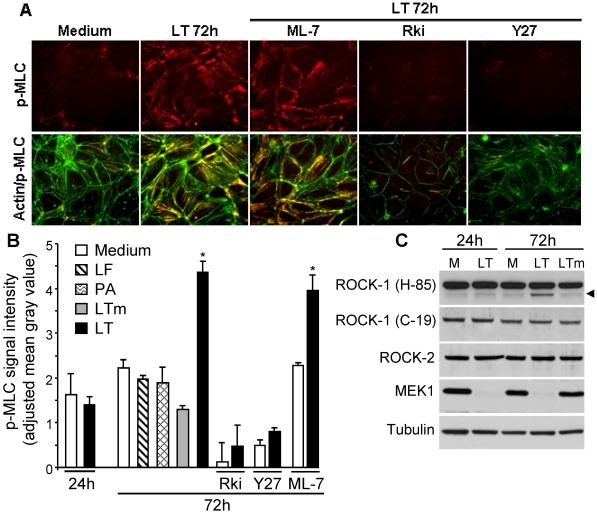
LT enhancement of myosin light chain (MLC) phosphorylation is attenuated by Rho-associated kinase (ROCK) inhibition. Cells were treated with medium, lethal factor (LF) (100 ng/mL), protective antigen (PA) (500 ng/mL), LT (100 ng/mL LF + 500 ng/mL PA), or inactive mutant LT (LTm; 100 ng/mL LF_E687C_ + 500 ng/mL PA). Where indicated, cells were pretreated with the MLCK inhibitor ML-7 (5 µM), or the ROCK inhibitors Rki (1 µM) or Y27 (5 µM) for 30 min. (**A**) After 72 h, monolayers were stained with a p-MLC antibody (red) and phalloidin (green, actin). Immunofluorescence images (400× magnification) are representative of four separate experiments. (**B**) Fluorescence signal intensity of p-MLC. Means ± SE are shown for at least three separate experiments except for ML-7 and Y27 alone (*n* = 2). * *p* < 0.001 *versus* medium. (**C**) Western blot of ROCK-1 (160 kDa), ROCK-2 (160 kDa), and MEK1 (45 kDa) at 24 and 72 h. ROCK-1 (H-85) recognizes an internal region whereas ROCK (C-19) recognizes the *C*-terminal end of ROCK-1. Arrowhead depicts a cleavage band of ROCK-1. Blots are representative of three experiments. Tubulin served as a loading control. M: medium.

We previously showed that LT-induced changes in AJs were accompanied by actin stress fibers [22]. Actin stress fibers are typically triggered by myosin light chain phosphorylation (p-MLC) that leads to extensive cross-linking of myosin and actin. To examine the relationship between p-MLC and LT stress fibers, we analyzed the effect of LT on p-MLC using semi-quantitative immunofluorescence. Non-treated cells showed low levels of p-MLC that were mainly associated with the cortical actin cytoskeleton (Figure 2A). At 72 h, LT-treated cells showed a significant two-fold increase in p-MLC signal intensity that co-localized with actin stress fibers (Figure 2A,B). Treatment with LF, PA, or mutant LTm did not increase p-MLC (Figure 2B). No change in p-MLC signal intensity was observed in LT-treated cells at 24 h consistent with the absence of significant actin stress fibers at this time point (Figure 2B).

MLC phosphorylation is primarily catalyzed by myosin light chain kinase (MLCK) and ROCK [29]. To examine the involvement of each of these kinases, cells were pretreated with the MLCK inhibitor ML-7 or the ROCK inhibitors H-1152 (Rki) or Y-27632 (Y27) for 30 min prior to LT. LT-induced p-MLC was not attenuated by ML-7 but was dramatically reduced by Y27 or Rki (Figure 2A,B). Higher concentrations of ML-7 (20 μM) also failed to inhibit LT stress fibers and p-MLC enhancement (data not shown). Western blot analysis of MEK1 cleavage by Western blot confirmed that Rki or Y27 did not inhibit the cellular entry or activity of LT (data not shown). Notably, Rki and Y27 alone, but not ML-7, produced a significant reduction in basal p-MLC expression suggesting that constitutive ROCK activity may play a more important role than MLCK in maintaining basal MLC phosphorylation in this system (Figure 2B). While the present data suggest the involvement of the ROCK pathway, we cannot rule out the possibility that other pathways besides ROCK contribute to the formation of LT stress fibers.

To further delineate the contribution of ROCK, we analyzed the expression of the two main isoforms, ROCK-1 and ROCK-2. Total expression of both isoforms, each identified as a main band at 160 kDa, was similar in LT-treated cells compared to non-treated cells at 24 and 72 h (Figure 2C). At 72 h, a cleavage band was detected in LT-treated cells when using an antibody raised against an internal sequence of ROCK-1 (H-85). This cleavage fragment was not observed in non-treated cells or mutant LT-treated cells. Proteolytic cleavage of the inhibitory *C*-terminus domain of ROCK-1 has previously been shown to generate a constitutively active 130 kDa fragment [30,31,32]. To further examine the specificity of this putative cleavage band, we used a second ROCK-1 antibody (C-19) raised against the *C*-terminus of ROCK-1. This antibody recognized the 160 kDa band, but not the cleavage product in LT-treated cells. Notably, the cleavage band was not detected at 24 h suggesting a temporal correlation between the appearance of cleaved ROCK-1 and enhanced p-MLC. MEK1 cleavage analysis confirmed intracellular LT activity at both time points (Figure 2C). These data suggest the potential involvement of ROCK-1 in the maintenance of LT-induced stress fibers.

### 3.3. ROCK Inhibition Protects AJ Integrity

**Figure 3:**
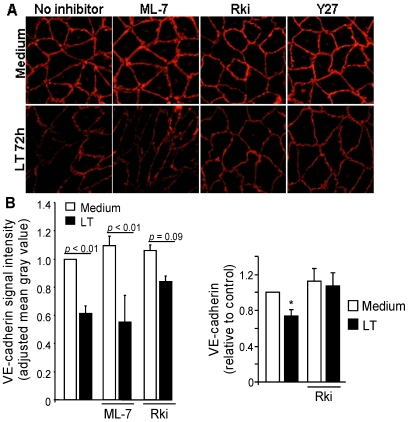
LT-induced VE-cadherin changes are attenuated by ROCK inhibition. Cells were treated with medium or LT. Where indicated, cells were pretreated with the MLCK inhibitor ML-7 (5 µM), or the ROCK inhibitors Rki (1 µM) or Y27 (5 µM) for 30 min. (**A**) After 72 h, monolayers were stained with a VE-cadherin antibody. Immunofluorescence images (400× magnification) are representative of three separate experiments. (**B**) Fluorescence signal intensity of VE-cadherin. Means ± SE for three separate experiments are shown. (**C**) Western blot analysis of VE-cadherin after 72 h. Densitometry analysis was performed for at least three separate experiments. Blots were reprobed for tubulin as a loading control. * *p* < 0.05 *versus* medium. Inh., inhibitor.

We hypothesized that modulating the formation of actin stress fibers could preserve VE-cadherin contacts. To test this hypothesis, we examined the effect of ROCK and MLCK inhibitors on VE-cadherin localization and expression. In non-treated cells, VE-cadherin localized to intercellular junctions exhibiting a continuous and intense cortical staining pattern (Figure 3A). After 72 h, LT induced cellular elongation with a discontinuous and less intense VE-cadherin staining pattern. Treatment with LF, PA, or mutant LTm did not disrupt VE-cadherin localization [22]. Treatment with the ROCK and ML-7 inhibitors alone did not alter VE-cadherin staining. LT-induced elongation and VE-cadherin disruption were attenuated by Y27 and Rki, but not by ML-7, though the cortical VE-cadherin band appeared slightly thinner than in non-treated cells (Figure 3A). The protective effect of Rki on VE-cadherin expression and localization was further demonstrated by Western blot and semi-quantitative analysis of VE-cadherin immunofluorescence (Figure 3B,C). 

**Figure 4:**
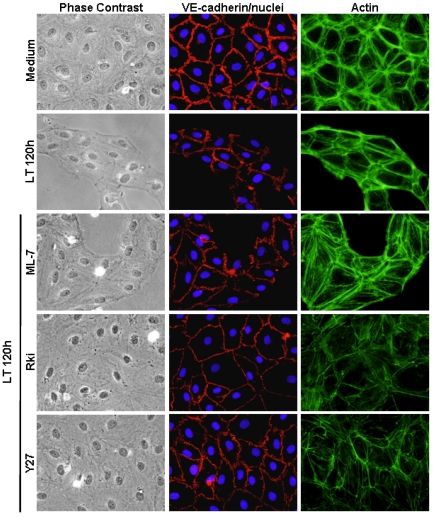
ROCK inhibition prevents long-term effects of LT on cellular architecture and VE-cadherin contacts. Cells were treated with medium or LT. Where indicated, cells were pretreated with the MLCK inhibitor ML-7 (5 µM), or the ROCK inhibitors Rki (1 µM) or Y27 (5 µM) for 30 min. After 120 h, monolayers were stained with Hoechst 33342 (blue, nuclei), phalloidin (green, actin), and a VE-cadherin antibody (red) as described in Materials and Methods. Representative fields are shown for each treatment by phase contrast and corresponding immunofluorescence images (400 × magnification).

In separate experiments, we investigated whether LT-induced AJ disruption could be ameliorated by inhibiting ROCK activity after LT exposure. Cells were treated with LT for 24 h prior to the addition of Y27. Similar to the inhibitor pretreatment experiments, Y27 prevented morphological changes and rescued VE-cadherin localization after 72 h (data not shown). The protective effect of ROCK inhibition on endothelial morphology and VE-cadherin contacts was particularly evident at time intervals beyond 72 h. After 120 h, LT-treated monolayers and VE-cadherin contacts were mostly disintegrated (Figure 4). Rki and Y27, but not ML-7, preserved monolayer integrity and VE-cadherin localization (Figure 4). While overall monolayer appearance was maintained by Rki and Y27, it is clear that the actin cytoskeleton remains dramatically altered compared to non-treated cells. Together, these data suggest that LT-induced cytoskeletal alterations involving the ROCK pathway may alter VE-cadherin expression and localization as a secondary event in response to the contractile phenotype. 

### 3.4. ROCK Inhibition Attenuates LT-Induced Albumin Leakage

**Figure 5:**
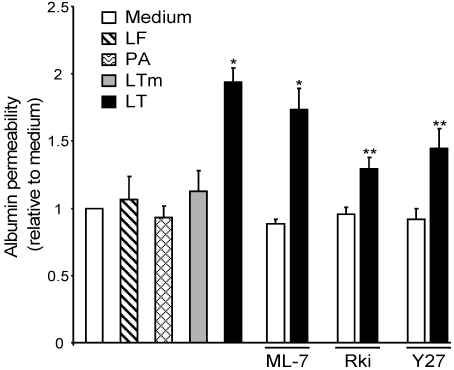
LT-induced barrier dysfunction is partially prevented by ROCK inhibition. Cells grown on porous membrane inserts were treated with medium or LT. Where indicated, cells were pretreated with the MLCK inhibitor ML-7 (5 µM), or the ROCK inhibitors Rki (1 µM) or Y27 (5 µM) for 30 min. After 72 h, albumin flux was measured as described in Materials and Methods. Means ± SE for three separate experiments are shown. * *p* < 0.001 *versus* medium; ** *p* < 0.01 *versus* LT.

We next examined whether the preservation of AJ integrity by ROCK inhibition translates to attenuation of barrier dysfunction. LT produced a two-fold increase in monolayer permeability to albumin. LF, PA, or mutant LTm had no effect on albumin flux. Rki and Y27, but not ML-7, significantly inhibited the LT-mediated increase in monolayer permeability to albumin (Figure 5). Together, these data confirm that actin stress fiber formation and AJ disruption play a prominent role in LT-mediated barrier dysfunction and suggest that the barrier can be protected, at least in part, by using ROCK inhibitors to block LT-induced morphological changes.

## 4. Discussion

Vascular leakage pathologies such as pleural effusion, edema, and hemorrhage are hallmarks of anthrax pathogenesis [8,9,10,11]. Animals treated with purified LT also succumb to vascular collapse, suggesting that the effects of LT on endothelium may play an important role in the pathophysiology of anthrax [12,20,21,33]. These observations are consistent with our previous findings that LT causes endothelial barrier dysfunction in a cell death-independent manner [22]. In the present study, we provide evidence that inhibition of the ROCK pathway can partially limit the increased monolayer permeability in LT-treated cells through stabilization of the AJ component VE-cadherin.

Endothelial AJs play a major role in regulating barrier function [34]. We found that LT caused a 40% decrease in VE-cadherin expression at 72 h, consistent with a reduction in VE-cadherin transcription and the altered immunofluorescence pattern and cell surface expression observed previously [22]. Interestingly, both the altered VE-cadherin expression and localization were attenuated by ROCK inhibition suggesting they may be secondary events in response to the contractile phenotype. The importance of the ROCK pathway in LT-induced barrier dysfunction was highlighted by the significant reduction in albumin flux in cells pre-treated with two separate ROCK inhibitors but not in cells pre-treated with an MLCK inhibitor. 

Due to the dynamic and selectively permeable nature of the endothelial barrier, several intrinsic signaling pathways function to modulate the barrier in cases where controlled passage of proteins and leukocytes is beneficial. Infectious and/or inflammatory agents often trigger these same mechanisms to induce pathological barrier dysfunction. One of these mechanisms involves rearrangement of the actin cytoskeleton from barrier-protective cortical actin to stress fibers, a common consequence of bacterial cytotoxins [35]. MLCK and ROCK are the main kinases that catalyze the phosphorylation of MLC, which stimulates myosin/actin cross-linking and results in stress fiber formation [29,31]. ROCK can also increase the amount of p-MLC by phosphorylating and inactivating MLC phosphatase. In the present study, we show that LT-induced morphological changes correlate with a two-fold enhancement in MLC phosphorylation. This enhancement and the LT-induced stress fiber formation, cellular elongation, and inter-endothelial gap formation were blocked by two ROCK inhibitors but not an inhibitor of MLCK.

While the present findings suggest a role for ROCK in LT-mediated endothelial dysfunction, the precise mechanisms have yet to be defined. An important experimental limitation in our study was that the ROCK inhibitors alone produced profound effects on the actin cytoskeleton. Moreover, under certain conditions, these inhibitors may nonspecifically inhibit other protein kinases such as PKA and PKC [31]. Thus, the present study does not rule out the involvement of other mechanisms, besides ROCK, as primary mediators of LT stress fibers. Indeed, Rolando and colleagues have reported that while ROCK is important for maintenance of LT-induced endothelial stress fibers, it does not appear that LT directly stimulates ROCK activity [36]. This conclusion was based, in part, on the observation that p-MLC was not enhanced in their study. This apparent inconsistency with our data may be related to differences in the cell type and time points investigated. Rolando *et al*. reported no differences in p-MLC in LT-treated cells over the course of 4-24 h. In our cell culture system, LT produced no changes in p-MLC after 24 h, but a two-fold enhancement after 72 h. 

The ROCK family consists of two closely related serine/threonine kinases, ROCK-1 and ROCK-2, both of which are highly expressed in the endothelium [31,37]. The two isoforms share an overall sequence homology of 65% at the amino acid level and a 92% homology within the kinase domain. Both ROCK isoforms can be activated through interaction with the GTP-bound form of RhoA or by proteolytic cleavage of their inhibitory C-terminal domain which results in a constitutively active truncated form. In the present study, we provide evidence for cleavage of ROCK-1, but not ROCK-2, in LT-treated cells. Cleaved ROCK-1 was detected at 72 h, but not 24 h, suggesting a temporal correlation with the LT-mediated enhancement of p-MLC. Previous studies have shown that ROCK-1, but not ROCK-2, is cleaved and activated by caspase-3 during apoptosis [30,31,32]. Caspase-independent mechanisms have also been reported [31,37]. Previously, we reported that caspase inhibition failed to prevent LT-mediated actin stress fibers and barrier dysfunction [22]. These previous findings are consistent with our recent preliminary data showing that caspase inhibitors failed to inhibit ROCK-1 cleavage and p-MLC enhancement in LT-treated cells. This also suggests the involvement of other caspase-independent cleavage mechanisms. 

Rolando and colleagues showed that LT does not activate the small GTPases RhoA, Rac and Cdc42 [36]. However, we have yet to assess the potential involvement of these upstream GTPases in our system. The potential contribution of other upstream pathways including PKC and PKA also warrant further investigation. The role of altered MAPK signaling also deserves greater attention, despite our previous findings showing that chemical MAPK inhibitors were unable to replicate LT-induced barrier dysfunction or cytoskeletal rearrangement [22]. Another permeability-enhancing mechanism reportedly triggered by VEGF and certain pathogens involves tyrosine phosphorylation of the AJ components by Src family tyrosine kinases [34,38,39]. This phosphorylation typically results in dissociation of the junctional components and reduced association with the actin cytoskeleton [40,41]. In separate experiments, LT did not enhance the tyrosine phosphorylation pathway, suggesting that this mechanism does not contribute to the observed barrier dysfunction. In addition, pre-treatment of cells with a Src inhibitor had no effect on morphological changes or enhanced albumin permeability triggered by LT (data not shown). 

Antibiotic therapy is currently the primary option to treat anthrax. However, antibiotics are not effective in treating the later stages of an infection due to substantial toxemia [42,43,44]. Toxin-directed therapies, including neutralizing antibodies as well as LF inhibitors are helpful but do not reverse established damage [17,45]. In light of the profound vascular pathologies associated with anthrax, the elucidation of the molecular mechanisms by which LT induces endothelial barrier dysfunction may help identify potential therapeutic targets to counter late-stage pathogenesis. In this regard, our finding that gross endothelial morphology changes and AJ disruption can be attenuated with a ROCK inhibitor treatment 24 h after LT exposure may be promising. Together, these data suggest that endothelium may be amenable to therapeutic intervention by targeting intrinsic barrier-regulating mechanisms.

## 5. Conclusions

Vascular leakage pathologies such as pleural effusion, edema, and hemorrhage are hallmarks of anthrax pathogenesis. Emerging evidence points to the endothelial lining of blood vessels as a critical pathogenic target. Anthrax LT, the major virulence factor of anthrax, triggers human endothelial barrier dysfunction *in vitro*. Here, we show that inhibition of ROCK ablates LT-induced morphology changes and dysregulation of AJs, a major barrier-regulating structure. Importantly, ROCK inhibition also provided significant protection against LT-induced barrier dysfunction. Together, these findings provide important insight into the profound endothelial dysfunction associated with anthrax and may be helpful in designing vascular-targeted therapies to halt disease progression and prolong the therapeutic window for countering the bacteremia.
